# Enhancing anticancer activity of checkpoint immunotherapy by targeting RAS

**DOI:** 10.1002/mco2.10

**Published:** 2020-06-25

**Authors:** Antonio B. Ward, Adam B. Keeton, Xi Chen, Tyler E. Mattox, Alex B. Coley, Yulia Y. Maxuitenko, Donald J. Buchsbaum, Troy D. Randall, Gang Zhou, Gary A. Piazza

**Affiliations:** ^1^ Drug Discovery Research Center Department of Pharmacology, Mitchell Cancer Institute University of South Alabama Mobile Alabama; ^2^ Department of Radiation Oncology University of Alabama at Birmingham Birmingham Alabama; ^3^ Department of Medicine, Division of Clinical Immunology and Rheumatology University of Alabama at Birmingham Birmingham Alabama; ^4^ Georgia Cancer Center Augusta University Augusta Georgia

**Keywords:** immunotherapy, PD‐L1, RAS, RAS inhibitor, tumor microenvironment

## Abstract

Approximately 30% of human cancers harbor a gain‐in‐function mutation in the RAS *gene*, resulting in constitutive activation of the RAS protein to stimulate downstream signaling, including the RAS‐mitogen activated protein kinase pathway that drives cancer cells to proliferate and metastasize. RAS‐driven oncogenesis also promotes immune evasion by increasing the expression of programmed cell death ligand‐1, reducing the expression of major histocompatibility complex molecules that present antigens to T‐lymphocytes and altering the expression of cytokines that promote the differentiation and accumulation of immune suppressive cell types such as myeloid‐derived suppressor cells, regulatory T‐cells, and cancer‐associated fibroblasts. Together, these changes lead to an immune suppressive tumor microenvironment that impedes T‐cell activation and infiltration and promotes the outgrowth and metastasis of tumor cells. As a result, despite the growing success of checkpoint immunotherapy, many patients with RAS‐driven tumors experience resistance to therapy and poor clinical outcomes. Therefore, RAS inhibitors in development have the potential to weaken cancer cell immune evasion and enhance the antitumor immune response to improve survival of patients with RAS‐driven cancers. This review highlights the potential of RAS inhibitors to enhance or broaden the anticancer activity of currently available checkpoint immunotherapy.

## Graphical abstract

**Inhibition of oncogenic RAS signaling increases antitumor immunity**. Inhibition of oncogenic RAS signaling results in an increase in antitumor immunity and tumor apoptosis due to an increase in cytotoxic T‐cell infiltration to the tumor site, a decrease in the immunosuppressive activity of TREGs and myeloid‐derived suppressor cells, and a decrease in tumor‐promoting cancer‐associated fibroblasts.

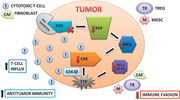

## 1 RAS AND CANCER PROGRESSION

A high percentage of human cancers harbor a gain‐in‐function mutation in RAS that results in the constitutive activation of either the KRAS, NRAS, or HRAS isozymes, which drive multiple aspects of malignant transformation and progression.^1^ Mutations in the KRAS isozyme are the most frequent of all cancers with RAS mutations.^2^ Cancers with the highest incidence of KRAS mutations are pancreatic cancer (90%), colorectal cancer (50%), and lung cancer (30%).^3^

As a small GTPase, RAS has a high affinity for guanine nucleotide binding and function as a molecular switch that cycles between an inactive guanosine diphosphate (GDP)‐bound and active guanosine triphosphate (GTP)‐bound state, in which the latter represents is responsible for interacting with effectors such as RAF or PI3K. Conversion between these two conformations are regulated by GTPase‐activating proteins (GAPs) and guanine nucleotide exchange factors (GEFs), respectively.^4^ Mutations in the RAS gene encode for RAS proteins with aberrantly slow GAP‐mediated GTPase cycling resulting in a profound increase in the activated form of RAS in a GTP‐bound state that drives tumorigenesis and metastasis.^5^, ^6^, ^7^ RAS signaling can bypass specific cell cycle checkpoints that control the entry and exit of cells into mitosis, thereby blocking cancer cell apoptosis to extend survival.^8^ In addition to mutations, RAS can be activated by growth factor stimulation or gain‐in‐function mutations in upstream signaling components, including various receptor tyrosine kinases.^9^

In addition to the widely appreciated role of activated RAS driving cancer cell signaling through binding to effector proteins such as the serine/threonine‐protein kinase, RAF, which can activate the mitogen activated protein kinase (MAPK) cell signaling pathway to drive cancer cell proliferation, constitutively activated RAS is also now recognized to suppress the body's natural defense mechanisms of immune surveillance.^10^ The role of RAS in blocking tumor immunity and its contribution to tumorigenesis is an emerging new field of research. Exploring the relationship that exists between the immune system and RAS activation has the potential to fully utilize the benefits of a RAS inhibitor as well as the capacity to broaden or enhance the activity of currently available checkpoint immunotherapy.

## 2 TARGETING RAS FOR CANCER TREATMENT

RAS‐driven cancers are generally the most lethal and unresponsive to chemotherapy and/or radiation and are therefore excluded from most therapies.^11^ In addition, individuals diagnosed with RAS mutant cancers have a lower life expectancy than those whose cancers are associated with other oncogenic driver mutations.^12^ A pan‐RAS inhibitor is expected to be an effective treatment for any RAS‐driven malignancies, including pancreatic, lung, and colorectal cancers^13^ and has the potential to be uniquely effective for the treatment of chemoresistant and/or radioresistant cancers.^14^, ^15^

Currently, there are no FDA approved drugs that directly inhibit RAS.^14^, ^16^ Until recently, RAS has often been said to be “undruggable” because of the high affinity of RAS to bind its substrate, GTP; high intracellular concentrations of GTP; and lack of suitable pockets on RAS amenable for small molecule binding.^17^ Nonetheless, a pan‐RAS inhibitor would be expected to have broad therapeutic benefits.^18^ Recent efforts to develop direct‐acting RAS inhibitors have largely focused on the synthesis of irreversible covalent inhibitors having reactive groups that target a cysteine residue present in the G12C mutant form of KRAS. Results from early clinical trials of two covalent KRAS G12C inhibitors, AMG‐510 and MRTX849, indicate that both are well‐tolerated and have antitumor activity for patients diagnosed with KRAS G12C mutant lung cancer.^19^ If proven to be effective in further clinical trials, mutation‐specific RAS inhibitors would be well suited for patients with a G12C RAS mutation that is present in approximately 13% of patients with lung adenocarcinomas.^20^

Given the higher prevalence of mutations other than G12C and the role of other RAS isozymes, which are coexpressed in cancer cells with a KRAS mutation, there remains a need for a pan‐RAS inhibitor that is not be limited to a specific RAS mutational codon or RAS isozyme. Such a drug would be expected to be effective for a broader range of RAS‐driven malignancies with potential for greater efficacy to inhibit both wild‐type (WT) and mutant RAS isozymes. A reversible pan‐RAS inhibitor would also have the potential for fewer side effects compared with covalent inhibitors with a higher likelihood of off‐target effects. Thus, RAS is of immense importance for many cancers with virtually thousands of laboratories studying various aspects of RAS biology and/or attempting to develop RAS inhibitors that target various vulnerabilities in cancer cells harboring RAS mutations.^21^ Insights gained from how RAS interacts with effectors and cycles from an inactive to an active conformation have provided novel approaches for small molecules that inhibit RAS in a reversible manner.^13^, ^22^ Perhaps there is no greater unmet medical need or opportunity in the field of oncology than for an efficacious and safe pan‐RAS inhibitor that acts in a reversible manner for the treatment of RAS‐driven malignancies alone or in combination with checkpoint immunotherapy.

## 3 RAS AND ANTITUMOR IMMUNITY

Cancer cells left unregulated by the immune system have the potential to form tumors that can metastasize to other organs in the body. Cancer progression is due to the ability of cancer cells to grow uncontrolled, metastasize, and turn off the body's antitumor immune response.^23^ Failure of the immune response plays a major role in the progression of cancer in which RAS is known to activate mechanisms of immune suppression.^24^ Previous research has linked RAS‐mediated signaling with modulation of cancer cell immunity.^25^ It is hypothesized that constitutively activated RAS signaling may be responsible for creating a “cold” tumor microenvironment (TME) devoid of immune cells that would otherwise suppress tumor growth. In support of this hypothesis, inhibition of activated RAS signaling by the G12C RAS mutant‐specific inhibitor, AMG‐510, alone or in combination with checkpoint immunotherapy increased antitumor efficacy in an immunocompetent animal model compared with an immune deficient model, and led to a proinflammatory or “hot” TME composed of immune cells with high anticancer activity in immunocompetent mice implanted with mutant RAS colorectal tumor cells.^20^ Other studies showed that the administration of the MEK inhibitor, trametinib, alone or in combination with checkpoint immunotherapy led to an increase in tumor‐infiltrating lymphocytes (TILs) with high anticancer activity, therefore creating a “hot” TME.^26^ Oncogenic RAS signaling is thought to activate mechanisms of immune suppression that allow cancer cells to evade the antitumor immune response.^24^ RAS signaling can render cancer cells undetectable by regulating immune cells such as TILs in the TME.^27^ Studies has reported that RAS activation in cooperation with other oncogenic drivers such as MYC cause the transition of lung adenomas to highly proliferative and invasive lung adenocarcinomas in vivo due to immune suppression of the TME.^28^ Cancer cell immune evasion results from a decrease in immune cell tumor infiltration due to an increase in activation of cancer cell immune checkpoint molecules on the surface of cancer cells.^29^ Inhibiting oncogenic RAS signaling has the potential to enhance the immune response in eradicating cancer cells by activating surveillance mechanisms of anticancer immunity.

## 4 RAS AND IMMUNE CHECKPOINT CELL SIGNALING

**FIGURE 1:**
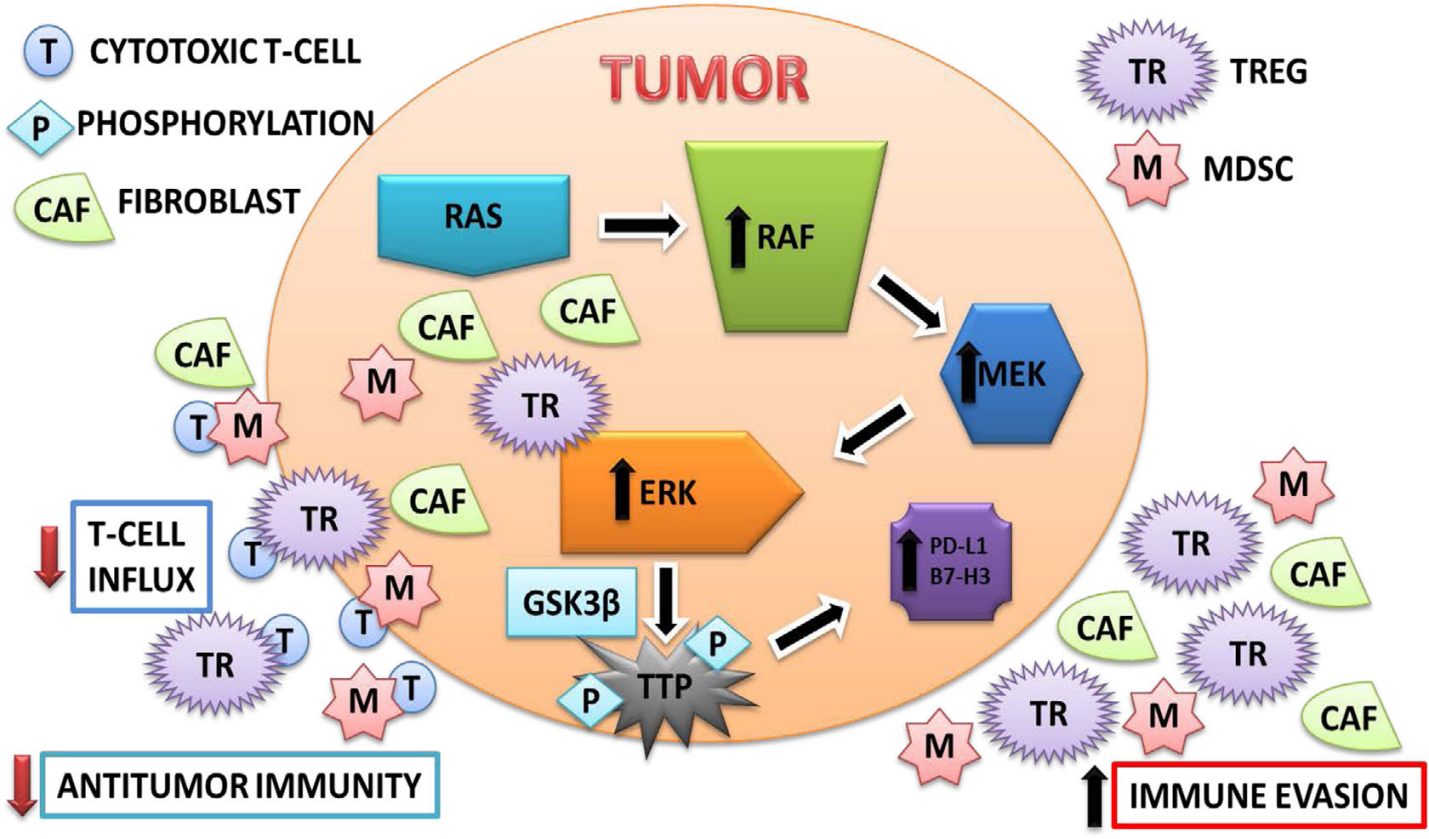
Oncogenic RAS signaling increases tumor immune evasion. Oncogenic RAS signaling results in an increase in tumor immune evasion and tumor survival due to a decrease in cytotoxic T‐cell infiltration to the tumor site, an increase in the immunosuppressive activity of TREGs and MDSCs within the TME, and an increase in tumor promoting CAFs.

RAS‐driven cancers frequently express ligands called immune checkpoint molecules on their surface that bind their cognate receptors on immune cells of the TME including CD4 helper T‐cells and CD8 cytotoxic T‐cells, as well as natural killer cells resulting in their functional exhaustion and decreased ability to kill cancer cells. T‐cells are activated by recognizing tumor antigen‐derived peptides in association with major histocompatibility complex (MHC) molecules presented by antigen presenting cells such as dendritic cells, macrophages, and B‐cells. The activated T‐cells (ie, effector T‐cells) then can recognize the relevant peptide ligands displayed by MHC molecules on tumor cells and exert tumor killing activities via various mechanisms, including secretion of cytokines such as interferon‐gamma (IFNγ) and tumor necrosis factor‐alpha (TNFα), release of cytotoxic granules such as perforin and granzyme B, and expression of apoptosis‐inducing ligands such as apoptosis antigen‐1 (FAS) and TNF‐related apoptosis‐inducing ligand (TRAIL).^30^, ^31^, ^32^ However, RAS‐activated cancers exhibit an increase in immune checkpoint molecules on their surface that bind to T‐cells and inhibit their function, while simultaneously exhibiting a decrease in MHC molecules on their surface which contributes to tumor cell immune evasion.^33^ Specifically, expression of high levels of the immune checkpoint molecules programmed cell death ligand‐1 (PD‐L1) and B7‐H3, which play a role in cancer cell immune evasion are regulated by RAS signaling.^10^, ^34^, ^35^ PD‐L1 and B7‐H3 are transmembrane protein ligands expressed on the surface of cancer cells which suppress T‐cell activity in the TME by binding to the immune cell receptors, programmed cell death receptor‐1 (PD‐1) and cytotoxic T‐lymphocyte‐associated protein‐4 (CTLA‐4), on T‐cells to transmit inhibitory signaling to disable effector T‐cells, enabling tumor immune escape and progression.^36^, ^37^, ^38^ RAS mutations and high PD‐L1 levels and/or high B7‐H3 levels are associated with poor prognosis and survival in patients treated with conventional anticancer therapies.^35^, ^39^, ^40^ Oncogenic RAS signaling increases tumor cell surface expression of PD‐L1 and/or B7‐H3 by a mechanism that promotes an increase in mRNA stability by modulation of the AU‐rich element‐binding protein tristetraprolin (TTP).^41^ TTP is an mRNA‐binding protein that regulates the transcription, translation, and degradation of multiple proteins expressed in cancer.^42^, ^43^, ^44^ Constitutive activation of RAS and MAPK signaling results in the phosphorylation and inhibition of TTP by glycogen synthase kinase‐3 beta (GSK3β) leading to increased PD‐L1/B7‐H3 transcription and protein translation, and cell surface expression, resulting in inhibition of T‐cell‐mediated antitumor immunity (Figure 1).

In addition to the RAS‐driven expression of PD‐L1and B7‐H3 on tumor cells, oncogenic RAS often impairs the expression of antigen presentation by suppressing MHC expression.^45^, ^46^, ^47^ This phenomenon can be attributed, in part, to the inactivation of interferon (IFN) signaling,^25^, ^48^ which normally promotes MHC expression.^49^ Oncogenic RAS impairs IFN signaling in tumor cells by decreasing the expression of signal transducer and activator of transcription‐1 (STAT1), STAT2, and interferon regulatory factor‐1 (IRF1),^48^, ^50^, ^51^ signaling molecules important for IFN responsiveness. Given that IFNγ signaling is an important pathway used by T‐cells to kill tumor cells,^52^ the RAS‐mediated reduction of IFNγ signaling likely promotes immune escape. Thus, RAS signaling is not only involved with intrinsic tumor cell proliferation and metastatic potential but also plays an important role in cancer cell immune evasion.

## 5 RAS REGULATION OF THE TME

Oncogenic RAS signaling in cancer cells regulates the activity of immune cells in the TME.^24^, ^53^, ^54^ The TME is composed of a network of cells that surround a tumor including T‐cells, fibroblasts, macrophages, and myeloid‐derived suppressor cells (MDSCs), all of which can influence tumor progression.^55^ As part of this process, oncogenic RAS alters the expression of cytokines and chemokines, including C‐X‐C motif chemokine ligand‐10 (CXCL10), interleukin‐10 (IL‐10), and transforming growth factor‐beta (TGF‐β), thereby impacting the recruitment, proliferation, and differentiation of T‐cells.^54^, ^56^, ^57^ For example, CD4 T‐cells responding to tumors with oncogenic RAS often differentiate into forkhead box P3 (FOXP3)‐expressing regulatory T‐cells (Tregs),^51^ likely due to increases in IL‐10 and TGF‐β expression by tumor cells.^26^ In turn, Tregs impair the proliferation and function of effector CD4 and CD8 T‐cells^58^ and potently suppress antitumor immunity. Oncogenic RAS also upregulates granulocyte‐macrophage colony‐stimulating factor (GM‐CSF), a cytokine that supports the accumulation of MDSCs, which suppress T‐cell function.^59^ Thus, oncogenic RAS uses multiple mechanisms, in addition to upregulating immune checkpoint molecules, to disrupt local T‐cell responses.

Oncogenic RAS signaling promotes the transformation of noncancerous stromal cells of the TME including fibroblasts into cancer associated fibroblasts (CAFs) by increasing the secretion of cytokines from cancer cells such as TGF‐β that promote the differentiation of fibroblasts into CAFs.^60^ These CAFs in turn secrete chemokines including vascular endothelial growth factor (VEGF) that increase cell signaling mechanisms that favor cancer cell progression, immune evasion, and tumorigenesis.^61^ CAFs of the TME promote an increase in PD‐L1 expression in cancer cells by secretion of the chemokine CXCL5 which induces the RAS‐MAPK signaling pathway.^62^ This paracrine effect between cancer cells and CAFs leads to an increase in overall RAS activation.^63^ These effects of RAS signaling on CAFs, Tregs, and MDSCs occur in concert with inactivation of T‐cells in the TME.^64^ Inhibiting oncogenic RAS signaling has the potential to alter the phenotypic profile of immune cells of the TME favoring an increase in anticancer activity and cancer cell apoptosis.

## 6 IMMUNOTHERAPEUTIC BENEFITS OF TARGETING RAS

**FIGURE 2:**
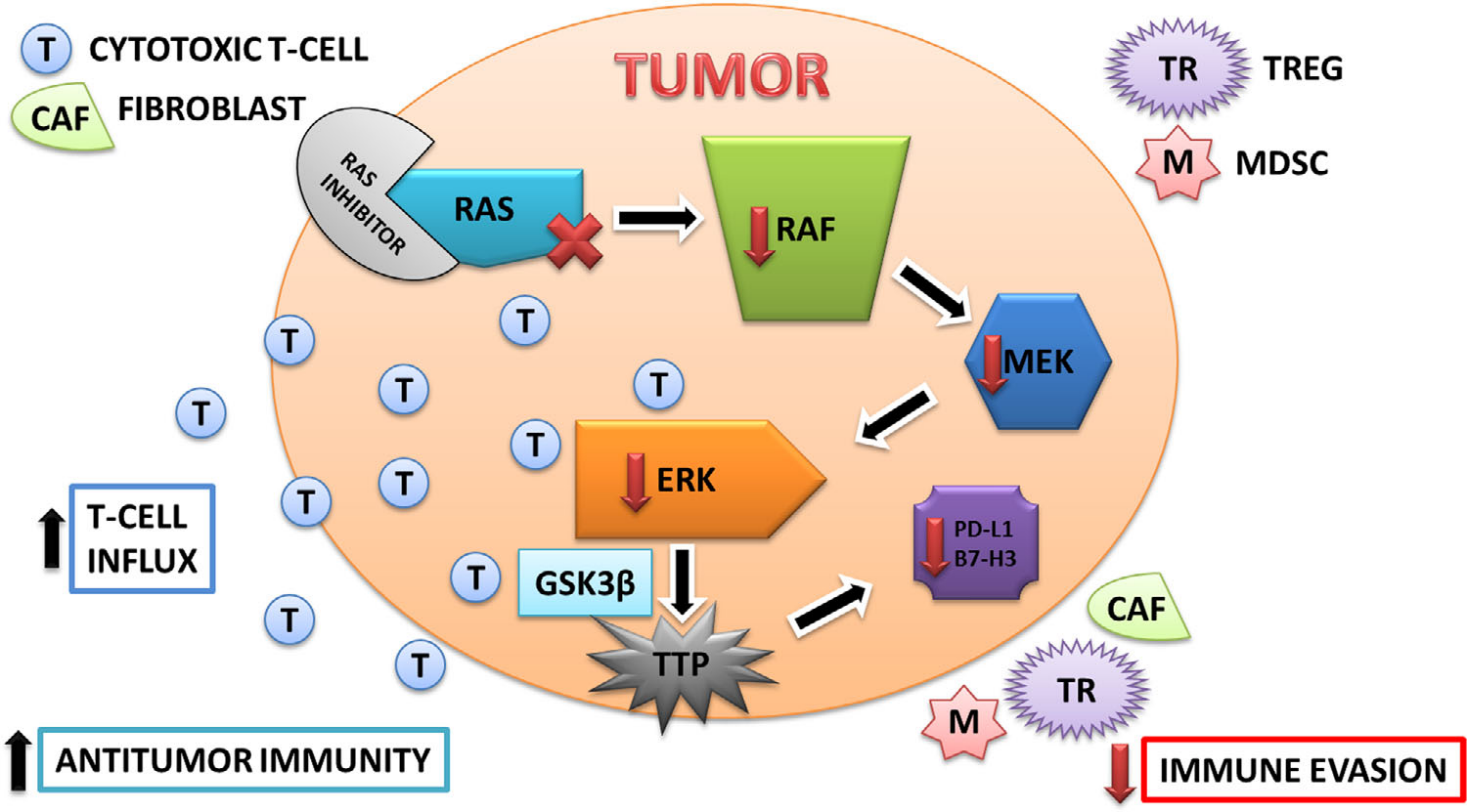
Inhibition of oncogenic RAS signaling increases antitumor immunity. Inhibition of oncogenic RAS signaling results in an increase in antitumor immunity and tumor apoptosis due to an increase in cytotoxic T‐cell infiltration to the tumor site, a decrease in the immunosuppressive activity of TREGs and MDSCs, and a decrease in tumor promoting CAFs.

In immunocompetent individuals, the immune system detects and destroys cancer cells by activating or turning on immune cells such as T‐cells.^65^ Tumors with a paucity of infiltrating T‐cells, such as RAS‐driven tumors, have high surface levels of immune checkpoint molecules such as PD‐L1 and B7‐H3 and respond poorly to current cancer treatments such as chemotherapy and immunotherapy.^35^, ^66^ Checkpoint immunotherapy assists and/or stimulates the immune system to suppress tumorigenesis and has become a promising new approach for difficult to treat cancers with early success being achieved to treat certain cancers such as melanoma.^67^ Immune checkpoint immunotherapy employs monoclonal antibodies to target immune checkpoint molecules such as PD‐1 and CTLA‐4 on T‐cells and their ligands PD‐L1 and B7‐H3 on tumor cells, restoring T‐cell anticancer activity.^36^, ^68^ Indeed, checkpoint immunotherapy is a unique and innovative method for cancer treatment, although there are limitations due to resistance and an overall modest success rate among the currently available FDA‐approved immunotherapies.^69^, ^70^ Targeted therapies that can possibly be used in synergy with immunotherapy drugs are needed for the treatment of aggressive tumors in cancer patients with RAS mutations.^71^, ^72^ Previous findings connecting oncogenic RAS activation with increased PD‐L1 levels in vitro and in vivo suggests potential benefits of combining targeted therapy using a small molecule inhibitor of RAS with immunotherapy using immune checkpoint inhibitors or monoclonal antibody blockade of PD‐1, CTLA‐4, B7‐H3, and PD‐L1 thereby blocking the immune checkpoint activity of cancer cells and increasing the anticancer activity of immune cells, especially T‐cells.^23^, ^35^, ^36^, ^73^, ^74^, ^75^, ^76^, ^77^ For example, treatment with a G12C inhibitor of RAS combined with anti‐PD‐1 therapy led to enhanced T‐cell tumor infiltration, activation, and an increase in tumor cell killing, in a syngeneic mouse model of colorectal cancer.^20^ The combination of a serine/threonine‐protein kinase B‐raf (BRAF) inhibitor with anti‐CTLA‐4 therapy resulted in slower disease progression and increased survival in melanoma patients compared to single agent therapy.^78^ Therefore, inhibiting oncogenic RAS signaling created a TME that was highly responsive to immune checkpoint inhibition or immunotherapy. A RAS inhibitor has the potential to stimulate the immune system by decreasing the RAS‐MAPK signaling pathway resulting in a decrease in PD‐L1 and/or B7‐H3 expression on the surface of cancer cells, which favors T‐cell antitumor activity (Figure 2). Therefore, combining small molecule targeted therapy, specifically a pan‐RAS inhibitor, with checkpoint immunotherapy should yield even greater therapeutic efficacy for a broad range of RAS‐driven cancers.

## 7 CONCLUSION

Gain‐in‐function mutations in the RAS *genes*, which activate signaling pathways essential for cancer cell proliferation, survival, and metastasis, are among the most common mutations driving a large percentage of human malignancies. RAS‐driven cancers are generally the most lethal and unresponsive to chemotherapy or radiation therapy. Promising reports from early clinical trials of the G12C mutation‐specific covalent inhibitors of KRAS support the concept of targeting RAS, which many have considered to be an “undruggable” target. The role of RAS in evasion of antitumor immunity by increasing the expression of immune checkpoint molecules has been less well studied, yet PD‐L1 and B7‐H3 are highly expressed in cancers harboring RAS mutations and associated with poor survival prognosis. Checkpoint immunotherapy is a powerful therapeutic modality with promising outcomes having been achieved for certain cancers such as melanoma, but many other cancers are refractory, often for reasons largely unknown. In addition, many patients ultimately relapse following an initial response. Reports support the hypothesis that broader and more efficacious anticancer activity can be achieved by combining checkpoint immunotherapy with a targeted therapy, particularly a RAS inhibitor. This would expand the scope of a RAS inhibitor for use not only as an important tumor‐directed therapeutic agent but also as part of a therapeutic strategy in combination with immunotherapy agents for RAS‐driven malignancies.

## CONFLICTS OF INTEREST

G.A. Piazza, A.B. Keeton, and X. Chen are cofounders of ADT Pharmaceuticals LLC and consultants.
